# A first immunohistochemistry study of transketolase and transketolase-like 1 expression in canine hyperplastic and neoplastic mammary lesions

**DOI:** 10.1186/s12917-017-0961-3

**Published:** 2017-01-31

**Authors:** Giovanni Pietro Burrai, Alessandro Tanca, Tiziana Cubeddu, Marcello Abbondio, Marta Polinas, Maria Filippa Addis, Elisabetta Antuofermo

**Affiliations:** 1 Department of Veterinary Medicine, University of Sassari, Via Vienna 2, 07100 Sassari, Italy; 2 Porto Conte Ricerche, S.P. 55 Porto Conte/Capo Caccia Km 8.400, Loc, 07041 Tramariglio, Alghero Italy

**Keywords:** Canine mammary tumors, Immunohistochemistry, Transketolase, Transketolase-like 1

## Abstract

**Background:**

Canine mammary tumors represent the most common neoplasm in female dogs, and the discovery of cancer biomarkers and their translation to clinical relevant assays is a key requirement in the war on cancer. Since the description of the ‘Warburg effect’, the reprogramming of metabolic pathways is considered a hallmark of pathological changes in cancer cells. In this study, we investigate the expression of two cancer-related metabolic enzymes, transketolase (TKT) and transketolase-like 1 (TKTL1), involved in the pentose phosphate pathway (PPP), an alternative metabolic pathway for glucose breakdown that could promote cancer by providing the precursors and energy required for rapidly growing cells.

**Results:**

TKT and TKTL1 protein expression was investigated by immunohistochemistry in canine normal (*N* = 6) and hyperplastic glands (*N* = 3), as well as in benign (*N* = 11) and malignant mammary tumors (*N* = 17). TKT expression was higher in hyperplastic lesions and in both benign and malignant tumors compared to the normal mammary gland, while TKTL1 levels were remarkably higher in hyperplastic lesions, simple adenomas and simple carcinomas than in the normal mammary glands (*P* < *0.05*).

**Conclusions:**

This study reveals that the expression of a key PPP enzyme varies along the evolution of canine mammary neoplastic lesions, and supports a role of metabolic changes in the development of canine mammary tumors.

**Electronic supplementary material:**

The online version of this article (doi:10.1186/s12917-017-0961-3) contains supplementary material, which is available to authorized users.

## Background

Cancer is the leading cause of death in companion animals, and mammary tumor, the most common neoplasm in female dog, represents a serious issue in worldwide veterinary practice [1, 2]. The etiopathogenesis of canine mammary tumors (CMT) is still unclear. Despite several authors reported genetic alterations of oncogenes and tumor suppressor genes, biological and morphological heterogeneity of CMTs has challenged veterinary pathologists since the early days of diagnostic pathology [3, 4].

As in humans, the identification of prognostic markers represents a major area of investigation in canine mammary cancer and an increasing number of potential prognostic factors, both clinicopathological and molecular, have been investigated [2, 4, 5]. However, classical clinicopathological features are not always sufficient to predict the biological behavior of CMTs. A few studies employed proteomic approaches to identify proteins related to the development and the aggressiveness of canine mammary tumors [6–9].

In a previous study performed by our group, several proteins showing an increased abundance in both tumor and non-tumor bearing canine mammary glands have been identified. We focused our attention on the enzyme transketolase (TKT) [9]. TKT, a dimeric protein composed of two monomers of about 68 kDa belonging to the family of transferases, is a thiamin diphosphate-dependent enzyme that catalyzes a reversible reaction by the transfer of two carbon atoms from an aldose to a ketose in the non-oxidative branch of the pentose phosphate/hexose monophosphate shunt pathway (PPP) [10–12]. The PPP pathway has been shown to generate *de novo* ribose-5-phosphate (R5P) and NADPH, and therefore is thought to play a major role in the proliferation of cancer cells [12, 13]. Constraining TKT activity, and consequently the PPP, using glycolytic pathway inhibitors such as oxythiamine or oxybenfothiamin, was shown to induce cell apoptosis and to inhibit cell proliferation by the reduction of the major RNA backbone, R5P, and the main antioxidant NADPH [14–17].

The human genome, in addition to TKT, encodes for two further TKT-related proteins, termed transketolase-like 1 (TKTL1) and transketolase-like 2 (TKTL2), which share 61 and 66% amino acid sequence homology with TKT, respectively [11]. A marked difference between TKT and TKTL1 is a deletion of 38 amino acids of the N-terminal catalytic domain in the latter, suggesting that TKTL1 is incapable of binding to the thiamine pyrophosphate and carrying out the TKT reaction [10, 17].

Furthermore, TKTL1 has been extensively investigated in cancers, and it has been supposed to be a catalytically active mutant form of human TKT, through formation of heterodimers with other TKT isoforms and/or by activation of other thiamine derivatives [13, 15].

TKTL1 is overexpressed in a range of human malignancies including breast, colon, ovary, lung, nasopharynx, gastric, renal, cervical, lung and liver cancers, and increased TKTL1 levels were shown to be associated with reduced survival for patients with cancers of the colon, oropharynx, bladder and with oral squamous cell carcinomas [18–24].

To date, no studies have characterized the protein expression of TKT and TKTL1 and their potential role in the onset of canine mammary tumors. Here, we investigate the expression of TKT and TKTL1 in canine mammary tissues by immunohistochemistry, exploring hyperplastic lesions as well as benign and malignant tumors, including simple and complex types.

## Methods

### Tissue collection

Thirty-seven fresh mammary samples were obtained from 35 female dogs that underwent surgery for mammary neoplasia at the Sassari Veterinary Hospital. Dogs belonged to the following breeds: Mixed breed (20), Yorkshire Terrier (7), German Shepherd (3) Dachshund (2), Pinscher (1) Labrador Retriever (1), Poodle (1). The dog ages ranged from 5 to 15 years (median 11.5 years).

Experiment permission was not required from the University’s Animal Care Ethics Committee because all the samples were retrieved from the archive of the pathology laboratory and were used for diagnostic purposes. Immediately after the surgical resection, the specimens were divided into two aliquots and stored in appropriate conditions based on the downstream analyses to be performed.

For histological examination, 10% formalin fixed samples were dehydrated in graded alcohol, embedded in paraffin wax, 3 μm-sectioned and stained with haematoxylin and eosin (H&E). Mammary samples were classified according to the World Health Organization criteria for canine mammary neoplasms [25]. In addition, canine mammary hyperplastic lesions were further evaluated as previously described by Antuofermo et al. and Mouser et al. [26, 27]. Lesions were imaged using Nikon Eclipse 80i and digital computer images were recorded with a Nikon Ds-fi1 camera.

Western immunoblotting analysis, carried out in order to validate antibody specificity, employed complementary tissues from 3 normal mammary glands and 3 simple tubulopapillary carcinomas that had been snap frozen upon collection and archived at −80 °C. After thawing, tissues were included in the Optimal Cutting Temperature medium (Tissue-Tek, Sakura Finetek, Torrance, CA, USA) and cut into 10 serial cryosections (Leica CM 1950, Heidelberg, Germany). Cryostat sections were histologically evaluated in order to confirm the presence of neoplastic lesions. Furthermore, HeLa cells (human fibroblasts derived from uterine cervix carcinoma) and ovine milk were retrieved to be used as positive and negative controls, respectively.

### Western immunoblotting

Proteins were extracted by incubating tissue and cell line samples in SDS-buffer as illustrated elsewhere [28]. Then, about 3 micrograms of each protein extract were separated by electrophoresis in a polyacrylamide gel (AnyKD, Bio-Rad, Hercules, CA, USA), blotted onto nitrocellulose membranes and blocked overnight with 3% bovine serum albumin (BSA) in phosphate-buffered saline (PBS) plus 0.05% Tween 20. TKT was detected upon sequential incubation with a mouse monoclonal anti-TKT primary antibody (1:10000 dilution in PBS plus 3% BSA and 0.05% Tween 20; clone ab112997, Abcam, Cambridge, UK) and a secondary antibody directed against mouse immunoglobulin (1:250000 dilution in PBS plus 1% BSA and 0.05% Tween 20; A9044, HRP, Sigma-Aldrich, Saint Louis, MO, USA). TKTL1 protein was detected upon sequential incubation with a rabbit polyclonal anti-TKTL1 primary antibody (1:2000 dilution in PBS plus 3% BSA and 0.05% Tween 20; clone LS-4019, LSBio, Seattle, WA, USA) and a secondary antibody directed against rabbit immunoglobulin (1:250000 dilution in PBS plus 1% BSA and 0.05% Tween 20; A9169, HRP, Sigma-Aldrich). The immunoreactivity was detected using a chemiluminescent peroxidase substrate (Sigma-Aldrich) and displayed with the VersaDoc Imaging System (Bio-Rad).

### Immunohistochemistry

To analyze the expression of TKT and TKTL1, histological sections (3 μm thick) from formalin-fixed, paraffin-embedded canine mammary tissue were mounted on positively charged Superfrost slides (Fisher Scientific). Slides were immersed for 20 min in a 98 °C preheated solution (WCAP, citrate pH 6, BiOptica, Milan, Italy) that simultaneously allows dewaxing, rehydration and antigen unmasking. Briefly, slides were mounted in a sequenza chamber (Shandon, Runcorn, UK) and tissues were then blocked for endogenous peroxidase with a 15 min incubation in Dako REAL Peroxidase-Blocking Solution (S2023, Dako, Glostrup, DK), and for nonspecific binding with 2.5% normal horse serum (ImmPRESS reagent kit, Vector Labs, Burlingame, CA, USA) for 30 min at room temperature.

Then, sections were incubated overnight at 4 °C with a mouse monoclonal anti-TKT antibody (clone ab112997, Abcam) and rabbit polyclonal anti-TKTL1 (clone LS-B4019, LSBio) at 1:150 and 1:100 dilution, respectively.

Then, sections were incubated for 20 min at room temperature with an anti-mouse/rabbit secondary antibody (MP-7500, ImmPRESS reagent kit, Vector Laboratories, Burlingame, CA, USA). 3,3′-Diaminobenzidine (DAB) (ImmPACT DAB, Vector Laboratories, Burlingame, CA, USA) was used as chromogen. All washing steps were performed three times with Tris-buffered saline (TBS) with 0.1% Tween 20 (BiOptica, Milano, Italy). Tissues were counterstained with haematoxylin, dehydrated and mounted with Eukitt Mounting Medium (BiOptica, Milan, Italy). A canine testis served as positive control, while negative controls were carried out by replacing the primary antibody with normal mouse or rabbit serum (ThermoFisher Scientific, Monza, Italy).

### Evaluation of immunohistochemical data

The extent of TKT immunopositivity was evaluated considering the nuclear signal, while the TKTL1 was quantified by estimating the cytoplasmic immunostaining. Immunoreactivity was semi-quantitatively scored considering the number of positive cells in 10 HPF (grade 0: no positive cells, 1: <10%; 2: 11–30%; 3: 31–60%; 4:> 60%) and the intensity of staining graded as weak (1), moderate (2), and strong (3). Then, a combined immunoreactivity score (IRS), ranging from 1 to 12, was calculated for each specimen by multiplying the values of these two categories.

The slides were reviewed independently by two authors (GPB, EA) and a consensus score was obtained for each case on a multiheaded microscope.

### Statistical Analysis

Statistical analysis of immunohistochemical expression data was carried out using nonparametric Kruskal-Wallis ANOVA followed by Dunn’s post hoc test. Spearman correlation analysis was performed to evaluate associations of TKT and TKTL1 protein levels. Data were analyzed with Stata version 11.2 (StataCorp, 2009), and results were considered significant when *P* ≤ *0.05*.

## Results

### Histological classification of lesions

Histologically, the lesions represent a morphologically heterogeneous group of samples and were classified as follows: normal mammary glands (*n* = 6), ductal hyperplasias (*n* = 3), benign tumors (*n* = 11; 6 simple adenomas and 5 complex adenomas), and carcinomas (*n* = 17; 11 simple tubulo-papillary carcinomas and 6 complex carcinomas). In addition, the 11 simple carcinomas were further characterized as well differentiated (2), moderately differentiated (5) and solid or undifferentiated type according to Pena and colleagues [29].

### Expression of TKT in canine mammary tissues

**Fig. 1:**
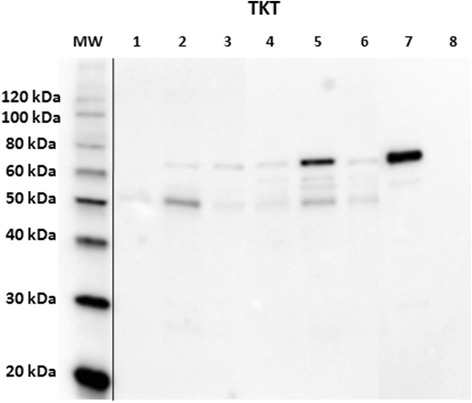
Western immunoblotting verification of anti-transketolase antibody reactivity in canine mammary tissues. *Lanes 1–3*: normal mammary glands; *lanes 4–6*: simple tubulo-papillary carcinomas. *Lane 7*: reactive control; *lane 8*: negative control; MW: molecular weight (MW) marker (MagicMark™ XP Western Protein Standard, Invitrogen). Standard MWs are displayed on the left side

Antibody reactivity was assessed on three normal mammary glands and three simple tubulo-papillary carcinomas (one well-differentiated and two moderately differentiated) by Western immunoblotting. Reactive bands were observed at the expected molecular weight range (around 65 kDa), with different signal intensities, in normal and in mammary tumor tissues, as well as in the positive control; no signal was detected in the negative control (Fig. 1). A band of lower molecular weight (approx. 50 kDa) was also present in mammary tumor samples, with different signal intensities, and was absent from both the positive and the negative control.

**Fig. 2:**
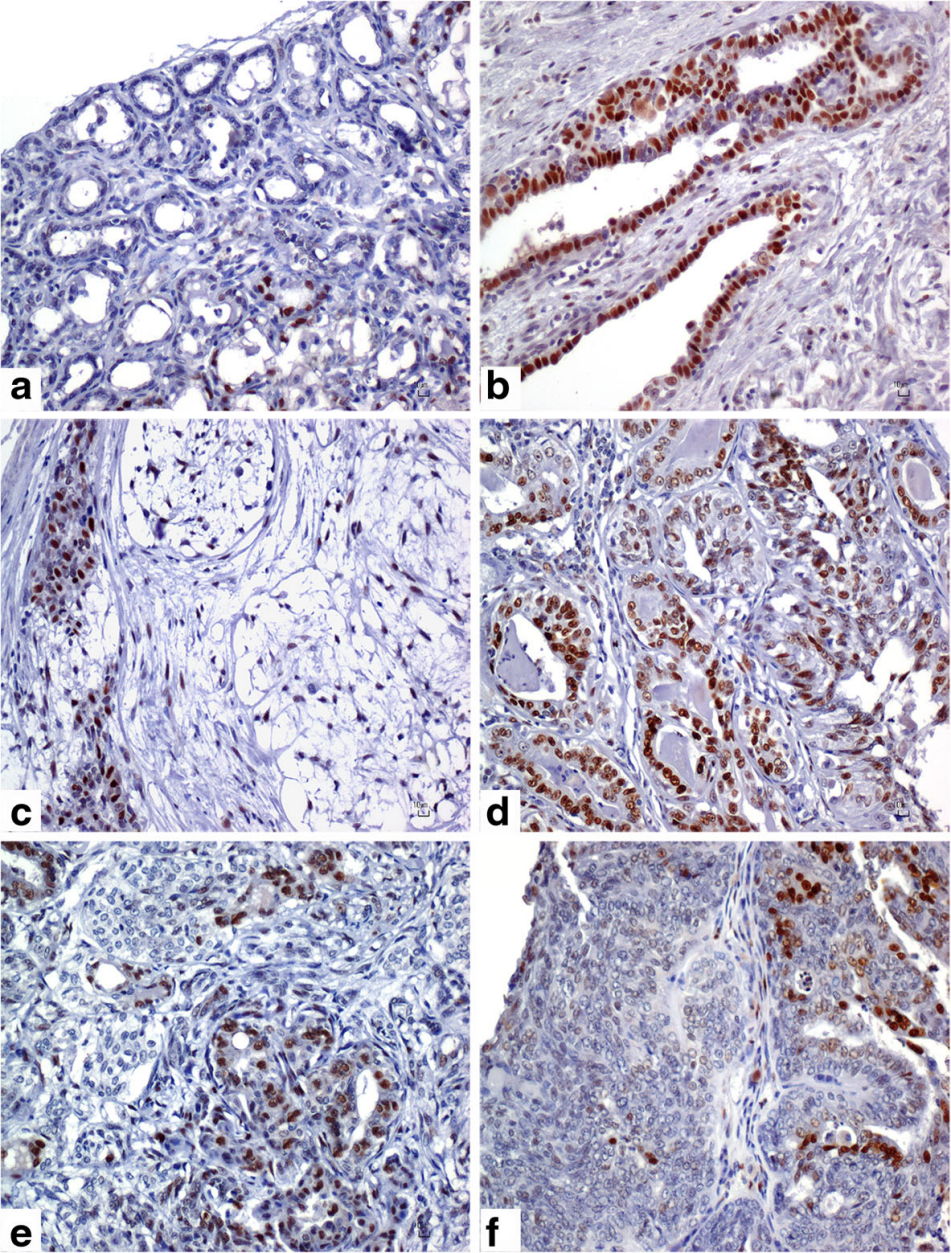
Transketolase immunohistochemical expression in canine mammary glands. Nuclear expression of TKT in epithelial cells of normal mammary gland (IRS = 1) (**a**), hyperplastic mammary gland (IRS = 9) (**b**), complex adenoma (IRS = 4) (**c**), simple adenoma (IRS = 6) (**d**), complex carcinoma (IRS = 4) (**e**) and simple carcinoma (IRS = 2) (**f**)

Upon immunohistochemical analysis, TKT expression was found in all tissue types, normal mammary glands (*n* = 4/6, 66%; IRS = 0, range 0–4), hyperplastic lesions (*n* = 3/3, 100%; IRS = 6, range 1–9), complex adenomas (*n* =5/5, 100%; IRS = 4, range 1–9), simple adenomas (n 6/6, 100%; IRS = 5, range 1–9) complex carcinomas (*n* = 6/6, 100%; IRS = 6, range 1–12) and simple carcinomas (*n* = 11/11, 100%; IRS = 1, range 1–9) (Fig. 2) (Additional file 1). Furthermore, statistical significant differences were noticed within the different histological lesions (Additional file 2). In comparison with normal mammary tissue, a significantly increased expression was observed in hyperplastic as well as neoplastic lesions, suggesting a pivotal role of TKT during the evolution of mammary carcinogenesis process (*P* < *0.001*).

In addition, a statistically significant difference between normal mammary gland and simple carcinomas (IRS =1) (*P* < *0.01*) was observed, corroborating the data obtained by mass spectrometry in our previous work, where TKT was found as differently expressed between tumor and normal gland both in formalin fixed and fresh frozen mammary tissues [9].

Moreover, simple carcinomas (IRS = 1) showed IRS values lower than hyperplastic lesions (IRS = 6) or benign and malignant tumors, both of simple and complex type, suggesting a possible existence of an additional pathway responsible for the onset of more aggressive mammary neoplasms (*P* < *0.05*). As a partial confirmation of this hypothesis, moderately differentiated carcinomas (grade II) showed lower IRS values compared to poorly differentiated simple carcinomas (grade III) (*P* < *0.05*).

### Expression of TKTL1 in canine mammary tissues

**Fig. 3:**
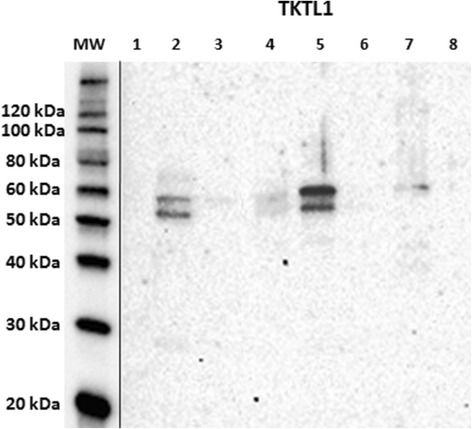
Western immunoblotting verification of anti-transketolase-like-1 antibody reactivity in canine mammary tissues. *Lanes 1–3*: normal mammary glands; *lanes 4–6*: simple tubulo-papillary carcinomas. *Lane 7*: reactive control; lane 8: negative control; MW: molecular weight (MW) marker (MagicMark™ XP Western Protein Standard, Invitrogen). Standard MWs are displayed on the left side

Antibody reactivity against TKTL1 was assessed as above, on the same 3 normal mammary glands and 3 simple tubulo-papillary carcinomas (1 well-differentiated and 2 moderately differentiated) by Western immunoblotting. Reactive bands were observed for TKTL1 at a slightly lower molecular weight (approx. 58 kDa) than TKT, with different signal intensities in normal and in mammary tumor tissues, as well as in the positive control; again, no signal was detected in the negative control. A further reactive band of lower molecular weight (approx. 53 kDa) was visible in canine mammary tissue samples, with different signal intensities, and was absent from the positive and the negative control (Fig. 3).

**Fig. 4:**
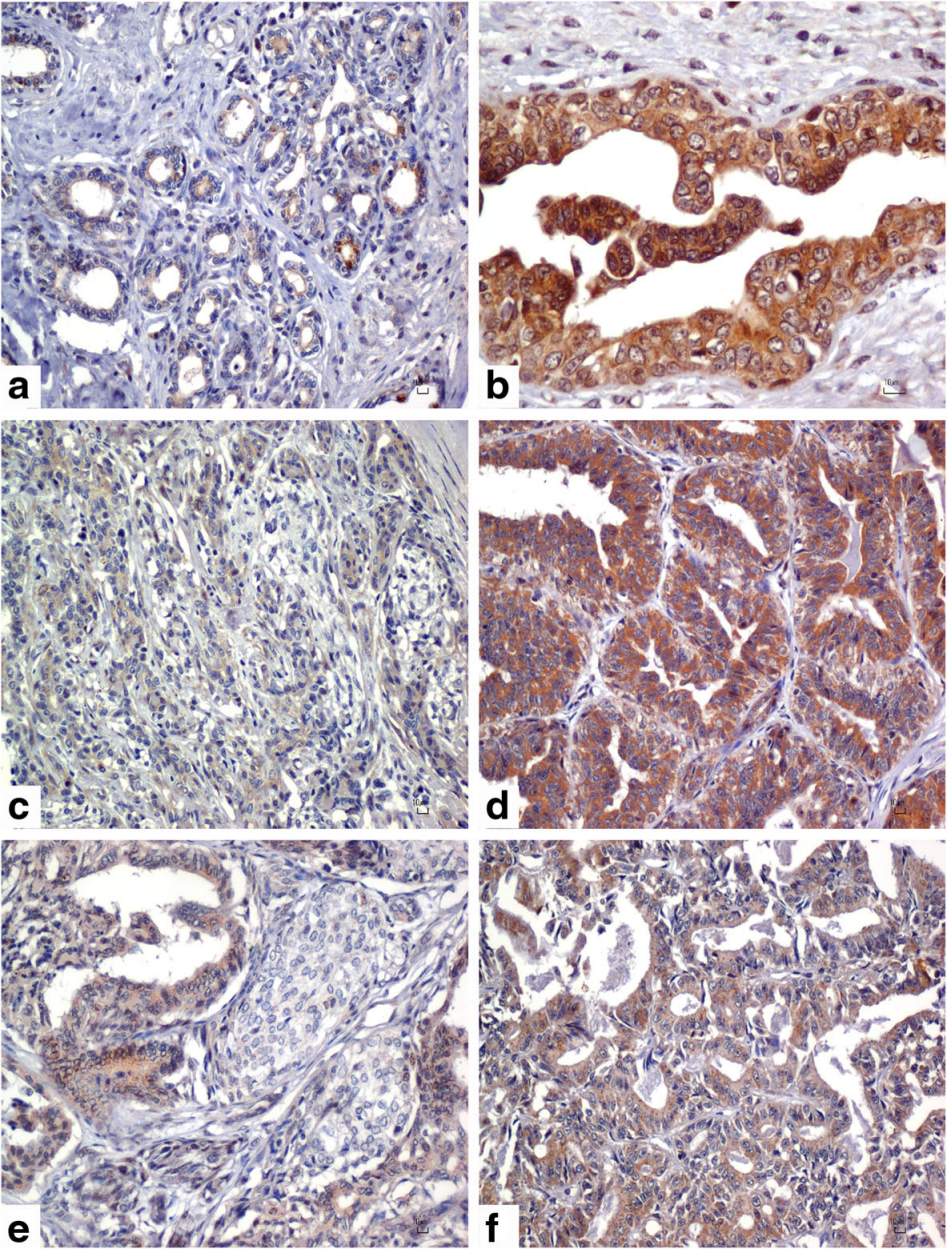
Transketolase like-1 immunohistochemical expression in canine mammary glands. Cytoplasmic expression in epithelial cells of normal mammary gland (IRS = 1) (**a**), hyperplastic mammary gland (IRS = 12) (**b**), complex adenoma (IRS = 1) (**c**), simple adenoma (IRS = 8) (**d**), complex carcinoma (IRS = 4) (**e**) and simple carcinoma (IRS = 6) (**f**)

Immunohistochemically, TKTL1 expression was found in all tissue types, normal mammary glands (*n* = 5/6, 83%; IRS = 1, range 0–12), hyperplastic lesions (*n* = 3/3, 100%; IRS = 12, range 9–12), complex adenomas (*n* = 5/5, 100%; IRS = 1, range 0–6), simple adenomas (*n* = 6/6, 100%; IRS = 8, range 1–12), complex carcinomas (*n* = 5/6, 83%; IRS = 1, range 0–12) and simple carcinomas (*n* = 11/11, 100%; IRS = 3, range 1–12) (Fig. 4) (Additional file 1).

In particular, hyperplastic lesions (IRS = 12), as well simple adenomas (IRS = 8) and simple carcinomas (IRS = 3), showed higher values than the normal mammary glands (*P* < *0.001*).

Of interest, hyperplastic lesions showed the highest values of TKTL1 expression when compared to all the other lesions, suggesting a possible role of the PPP in the early stage of neoplastic transformation (*P* = *0.001*).

Comparing the expression of TKTL1 among the various tumors, it is also worthy of note that the simple type adenomas and carcinomas tend to show a higher expression compared to the complex forms, whether they are benign (IRS = 1) and malignant (IRS = 1), indicating therefore a predominant role of TKTL1 in the epithelial cells compared to the myoepithelial component (*P* < *0.001*). This hypothesis is further supported by the higher TKTL1 expression in simple adenomas than in complex carcinomas (*P* < *0.001*) (Additional file 3).

Significant positive correlations were observed between TKT and TKTL1 expression in normal mammary gland (*ρ* = *0.59*, *P* < *0.05*), while a negative correlation was observed for complex adenomas (*ρ* = −*0.39*, *P* = *0.003*).

## Discussion

Reprogramming of metabolic pathways is considered a hallmark of pathological changes in cancer cells [30]. Since the description of the so-called ‘Warburg effect’, it has been known that neoplastic cells use the anaerobic degradation of glucose even in the presence of oxygen [31, 32].

This observation kept scientific focus on glycolysis and highlighted other aspects of glucose metabolism that could promote cancer by providing the precursors and energy required for a rapidly growing cell, such as adenosine 5′-triphosphate (ATP) and carbon intermediates [33].

Noteworthily, an alternative metabolic pathway for glucose breakdown is the pentose phosphate/hexose monophosphate shunt pathway [34].

The main purpose of the PPP is to generate R5P, which is used in the nucleotides synthesis, and NADPH, that neutralizes reactive oxygen species (ROS) enabling cancer cells to survive oxidative stress [35]. This can benefit cancer cells by facilitating cell proliferation, tumor invasion and resistance to apoptosis, which in turn promotes tumor invasion [36].

Thus, in this study, we sought to analyze by immunohistochemistry the expression of TKT, a key enzyme in the non-oxidative part of the PPP, and TKTL1, a TKT isoform frequently reported in human tumor related pathways, in a retrospective patient cohort with invasive canine mammary tumor as well as in normal and hyperplastic mammary glands. As a result, the expression of TKT was observed in all examined mammary tissues. Furthermore, the increased protein expression in hyperplastic lesions and in both benign and malignant tumors compared to normal mammary gland suggests a significant involvement of the PPP and, consequently a role of TKT during the canine mammary carcinogenesis process. The increased expression of TKT found in mammary tumors and in hyperplastic lesions of the dog appears to be substantially in agreement with those reported in recent studies carried out in human and veterinary medicine, in which there was an increased expression of TKT in cancer tissues and in progestin-induced canine mammary hyperplasia compared to non-tumor tissues, respectively [12, 36, 37].

In addition, our results lead to hypothesize that a pathway that synthesizes antioxidants, the PPP, is necessary to tumor development, particularly in hyperplastic lesions where TKT is an up regulated gene and reaches the highest protein expression values, thus confirming what has been described in the mouse mammary tumor model by Lu and colleagues [36, 38]. In fact, as elegantly proposed by Gatenby and Gilles in 2004, hyperplastic lesions are exposed to a hypoxic environment and acquire the glycolytic phenotype with the production of hydrogen ions (H^+^); this leads to the consequent acidification of the extracellular space, resulting in cellular toxicity due the increase of ROS [39]. Thus, an increase of PPP down-streaming molecules, such as NADHP, is necessary to counteract and mitigate oxidative stress, especially in hyperplastic lesions.

In this study, simple carcinomas showed a lower expression of TKT when compared to the others lesions, with a significant reduction of the enzyme in the most undifferentiated forms (i.e. simple carcinoma grade III). The underexpression trend of TKT in the more aggressive neoplasm suggests that probably a further molecular pathway exists that limits or inhibits the protein expression in the most aggressive and life-threatening mammary neoplastic histotype. For example, it was revealed that the tumor suppressor p53, often expressed in canine carcinomas, can inhibit the PPP by binding to glucose-6-phosphate dehydrogenase (G6PD), the rate-limiting enzyme in the oxidative branch of the PPP, which ultimately results in decreased transketolase expression [40, 41]. Moreover, additional and more focused studies are needed in order to confirm this hypothesis.

As a partial confirmation of the validity of our results and of the important role played by the TKT in the carcinogenic process, several studies report that the use of transketolase competitive inhibitors, such as oxythiamine, in experimental models significantly reduces the tumor cell proliferation rate [14, 16, 42].

Compelling evidence has proved that TKTL1 is a cancer related molecule and that it plays a key role in the onset of different neoplastic disease. Its overexpression predicts poor patient survival, tumor recurrence and resistance to chemo and radiation therapy in many cancers [18–24].

In our work, TKTL1 was expressed in most of the hyperplastic and neoplastic lesions and in 83% of non-neoplastic mammary tissue. Furthermore, the statistical analysis of different histological types showed an increased TKTL1 expression in hyperplastic lesions and in both benign and malignant simple tumors, in agreement with other reports in women breast cancer [18, 43]. However, in contrast to what described by these authors, TKTL1 expression in mammary tissues of the dog showed higher values in hyperplastic lesions and a reduction of the immunoreactivity as the degree of malignancy increased. This indicates that the protein could play a crucial role in the stepwise progression from early hyperplastic lesions to fully malignant CMT. In fact, hyperplastic lesions have been considered as risk factors for subsequent development of invasive mammary cancer both in human and in canine species [26, 44, 45]. In this perspective, we might speculate that the selective block of TKTL1 through the targeting of PPP metabolic pathway could limit neoplastic development in its early stages represented by hyperplastic and benign tumors, arresting tumor progression in its “*primum movens*”.

In addition, TKTL1 was more expressed in simple canine neoplasms, both benign and malignant, when compared to complex types, suggesting a predominant role of TKTL1 in epithelial cells *vs* the myoepithelial component. Moreover, to our knowledge, no studies have investigated the role of TKT or TKTL1 in myoepithelial cells, both in human and canine tumors, and consequently, definitive and conclusive hypothesis cannot be drawn.

## Conclusions

In conclusion, this is the first study investigating the expression of TKT and TKT-like enzymes in canine mammary tumors and hyperplastic lesions. We observed increased expression of TKT and TKTL1 in the proposed multistep carcinogenesis of CMT, thus indicating that PPP is a cancer metabolism-related pathway also in canine mammary tumors. TKTL1 has recently been used as a biomarker in a blood test based on the epitope detection in monocytes (EDIM) technology, allowing for the non-invasive detection of neoplasia and tumor recurrence, and thereby it has been proposed as a therapeutic target [42]. Similar efforts to identify important metabolic changes during canine mammary cancer progression hold the potential for providing putative diagnostic and prognostic biomarkers, as well as new therapeutic targets, also in canine species.

## Additional files

Additional file 1: Immunohistochemical evaluation of TKT and TKTL1. Immunoreactivity scores (IRS) of normal mammary glands (*n* = 6), ductal hyperplasias (*n* = 3), benign tumors (*n* = 11), and carcinomas (*n* = 17), considering the number and the stain intensity of positive cells per high power field (HPF). (XLSX 18 kb)
Additional file 2: Graphical representation (box-plot) of TKT immunohistochemical evaluation. Immunoreactivity scores (IRS) of normal mammary glands (*n* = 6), ductal hyperplasias (*n* = 3), benign tumors (*n* = 11) and carcinomas (*n* = 17), with statistical differences between lesions. Different letters (a, b, c, d) indicate significant differences (*P* < *0.05*), red line (median values), Kruskal-Wallis ANOVA followed by Dunn’s post hoc test. (TIF 1566 kb)
Additional file 3: Graphical representation (box-plot) of TKTL1 immunohistochemical evaluation. Immunoreactivity scores (IRS) of normal mammary glands (*n* = 6), ductal hyperplasias (*n* = 3), benign tumors (*n* = 11) and carcinomas (*n* = 17), with statistical differences between lesions. Different letters (a, b, c, d) indicate significant differences (*P* < *0.05*), red line (median values), Kruskal-Wallis ANOVA followed by Dunn’s post hoc test. (TIF 970 kb)

## Abbreviations

ATP: Adenosine 5′-triphosphate
BSA: Bovine serum albumin
CMT: Canine mammary tumors
EDIM: Epitope detection in monocytes
G6PD: Glucose-6-phosphate dehydrogenase
H&E: Haematoxylin and eosin
IRS: Immunoreactivity score
PBS: Phosphate-buffered saline
PPP: Pentose phosphate pathway
R5P: Ribose-5-phosphate
ROS: Reactive oxygen species
TKT: Transketolase
TKTL1: Transketolase-like 1
TKTL2: Transketolase-like 2
