# Supplementary figures and images for: A first immunohistochemistry study of transketolase and transketolase-like 1 expression in canine hyperplastic and neoplastic mammary lesions
Source: BMC Vet Res. 2017 Jan 31;13:38. doi: 10.1186/s12917-017-0961-3 (PMC5282725; doi:10.1186/s12917-017-0961-3)

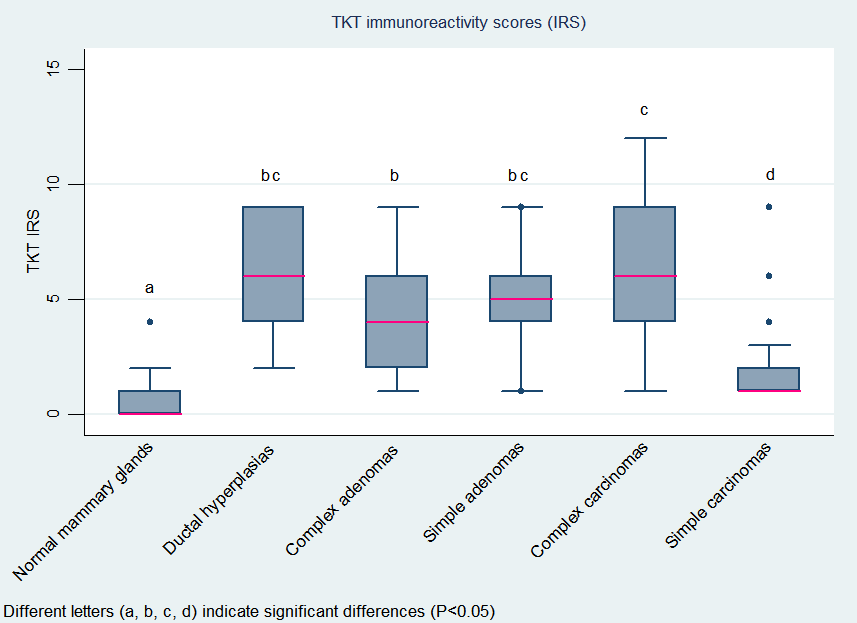

Supplement: Additional file 2: — Graphical representation (box-plot) of TKT immunohistochemical evaluation. Immunoreactivity scores (IRS) of normal mammary glands (n = 6), ductal hyperplasias (n = 3), benign tumors (n = 11) and carcinomas (n = 17), with statistical differences between lesions. Different letters (a, b, c, d) indicate significant differences (P < 0.05), red line (median values), Kruskal-Wallis ANOVA followed by Dunn’s post hoc test. (TIF 1566 kb) [file 12917_2017_961_MOESM2_ESM.tif]

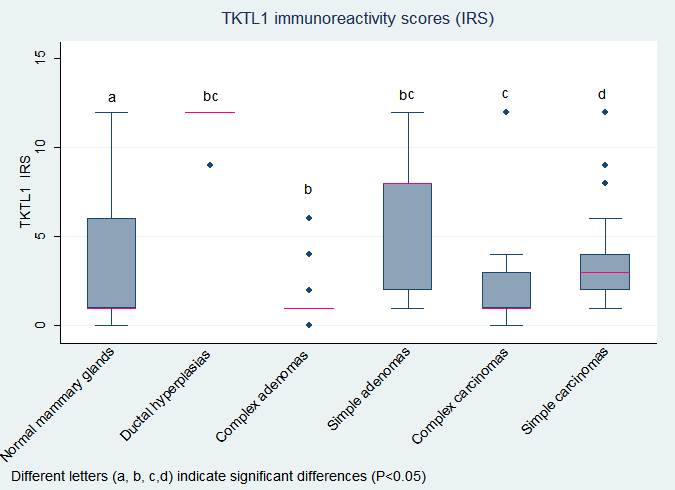

Supplement: Additional file 3: — Graphical representation (box-plot) of TKTL1 immunohistochemical evaluation. Immunoreactivity scores (IRS) of normal mammary glands (n = 6), ductal hyperplasias (n = 3), benign tumors (n = 11) and carcinomas (n = 17), with statistical differences between lesions. Different letters (a, b, c, d) indicate significant differences (P < 0.05), red line (median values), Kruskal-Wallis ANOVA followed by Dunn’s post hoc test. (TIF 970 kb) [file 12917_2017_961_MOESM3_ESM.tif]
